# Relationships between sedentary behaviour, physical activity levels and red blood cell distribution width in children and adolescents

**DOI:** 10.15171/hpp.2018.19

**Published:** 2018-04-18

**Authors:** Nevin Hammam, Victor E. Ezeugwu, Patricia J. Manns, Lesley Pritchard-Wiart

**Affiliations:** ^1^Department of Physical Therapy, Faculty of Rehabilitation Medicine, University of Alberta, Edmonton, AB, Canada; ^2^Rheumatology and Rehabilitation Department, Faculty of Medicine, Assiut University, Assiut, Egypt

**Keywords:** Epidemiology, Cardiovascular diseases, Accelerometer, Physical activity, Sedentary behaviour

## Abstract

**Background:** Red blood cell distribution width (RDW) is a biomarker for cardiovascular disease(CVD). RDW is associated with sedentary behavior (SB) and physical activity (PA) in adults.To date, no study has evaluated this association in children. The purpose of this study was to evaluate the association between RDW and SB and PA levels of children and adolescents.

**Methods:** This observational study included data from participants aged 12-20 years in the 2003–2006 National Health and Nutrition Examination Survey (NHANES). SB and PA were measured using accelerometers. Activity levels were classified into intensity categories. Sex specific multivariable regression analyses (adjusted for covariates) were used to explore the associations between SB, PA and RDW.

**Results:** The study included 2143 children and adolescents (1080 boys and 1063 girls). In the fully adjusted regression model for boys, SB was positively associated with RDW (β =0.116,P=0.004) while moderate PA was negatively associated with RDW (β =-0.082, P=0.048). In girls, there were no significant associations between activity levels and RDW.

**Conclusion:** This study provides preliminary evidence of the association between SB, moderate intensity PA and RDW in boys, but not in girls. Further research to determine the mechanisms associated with this relationship and underlying sex differences is warranted.

## Introduction

Cardiovascular disease (CVD) is the most common cause of mortality and morbidity worldwide,^1^ often manifesting clinically in middle or old age. Emerging findings, however, have demonstrated that some risk factors for CVD are present in childhood^2^ and that these factors are associated with an elevated lifetime risk for CVD.^3^ An attempt to discover the early predictors of CVD has provided the impetus for extensive work focused on the discovery of novel biomarkers and pathways in the early detection, prevention and management of CVD.^1^

Red blood cell distribution width (RDW), an indicator of anisocytosis, or increased variation in red blood cell (RBC) size, is a novel biomarker for CVD.^4^ Higher RDW is strongly associated with adverse clinical outcomes in patients with CVD,^5,6^ cerebrovascular disease,^7^ and is a predictor of all-cause CVD mortality. Although the pathophysiologic mechanism by which RDW is linked to CVD remains uncertain, most of the proposed mechanisms reflect an underlying inflammatory condition, oxidative stress or dyslipidemia.^8^ Since RDW has the potential to provide prognostic information about CVD risk without significant cost burden,^9^ there is interest in exploring the factors that alter RDW levels and, subsequently CVD risk.

It is well known that prolonged sedentary behavior (SB) and reduced physical activity (PA) are risk factors for CVD and major contributors to CVD mortality.^10-12^ There are several possible biological mechanisms by which increased PA contributes to reducing CVD risk including improved body composition, enhanced lipid metabolism,^13^ reduced blood pressure (BP)^14^ and reduced systemic inflammation.^15^ Specifically, C-reactive protein (CRP), a protein associated with chronic inflammation, is a strong predictor of CVD risk that can be decreased with increased PA and reduced SB.^16^ Exploring mechanisms linking SB, PA and CVD are warranted^17^ in order to refine screening strategies and to monitor the short-term effects of interventions on long-term CVD risk.

While there is evidence that increased RDW levels are associated with lower PA, higher SB, and the presence of CVD in adults,^9,18,19^ these relationships have not been explored in children and adolescents. If a similar association between RDW, SB and PA exist in children and adolescents, RDW may represent a potential target for CVD prevention. Identification of modifiable risk factors for CVD is important in the younger population before signs of CVD appear. In addition, changes in child PA behaviour may result in better PA habits in adolescence and ultimately in the adulthood.^20^

The purpose of this study was to describe the relationships between objectively measured SB, PA and RDW among children and adolescents, and to determine whether these relationships were independent of known factors affecting RDW.

## Materials and Methods

### Study design and participants

Data from the 2003–2006 National Health and Nutrition Examination Survey (NHANES) were selected for analyses. These cycles were the only ones with accelerometer data available at the time of data extraction and writing. The NHANES is an ongoing survey conducted by the Centers for Disease Control and Prevention to assess the health and nutrition status of a representative sample of adults and children in the U.S. Detailed NHANES study procedures can be found on the study website (https://www.cdc.gov/nchs/nhanes/). The 2 NHANES cycles (2003-2004 and 2005-2006) used in the current study included 20 470 children and adults. Participants aged 12-20 years, for whom complete data were available for the main study variables (i.e., SB, PA, and RDW) were selected for inclusion. The age range was in alignment with previous PA studies using NHANES data.^9,18,21^

### Measurement of physical activity and sedentary behavior

Ambulatory participants were instructed to wear a uniaxial accelerometer (ActiGraph 7164; ActiGraph LLC, Fort Walton Beach, Florida) for 7 days during all activities, with the exception of water based activities. Specific details on the NHANES accelerometer protocol are described elsewhere.^22^ Participants with valid activity data for at least 4 days with ≥10 hours per day were included in the analysis. Non-wear time was defined as ≥ 60 consecutive minutes of zero counts per minute (cpm), with the allowance of 1–2 minutes of counts between 0 and 100.^23^ The average time each participant spent per day in SB or different PA intensities was determined from the raw accelerometer counts and expressed as minutes per day (min/d) using the cut points recommended by Freedson et al.^24^ Activity counts ≤100 counts/min were considered as SB, 100- 2219 light PA (LPA), 2220–4135 moderate PA (MPA), and ≥4136 vigorous PA (VPA). Sedentary breaks, transition from sedentary (<100 cpm) to non-sedentary activity (≥100 cpm), sedentary bout length, consecutive minutes <100 cpm, active bout lengths, and consecutive active minutes ≥100 cpm were also calculated.

### Measurement of red blood cell distribution width

RDW, a component of the complete blood count analysis, was extracted from the NHANES database. RDW% represents variation of the red cell volume distribution and is reported in NHANES as a percentage.

### Covariates 

Covariates identified from previous research were selected for inclusion in the analysis.^17^ Sociodemographic information: age, sex, race-ethnicity variables were extracted from the NHANES data base. Exposure to smoke was determined by serum cotinine level. Socioeconomic status (SES) was assessed using family poverty income ratio (PIR); the ratio of poverty income to the federal poverty threshold. Anthropometric data: height, weight and waist circumference (WC) were measured by the NHANES team using standardized examination protocols. Body mass index (BMI) was calculated as weight divided by height squared (kg/m^2^). Clinical examination: BP was measured up to 4 consecutive times, and the average reported in mmHg. Dietary intake was measured using a 24-hour recall assessment of food and fluid for 2 days. Data were collected by a trained dietary interviewer. Total caloric intake over 2 days and proportions of caloric intake per specific food categories were calculated. Laboratory tests include complete blood count (CBC), hemoglobin (HGB) levels, serum CRP, serum uric acid, fasting blood glucose (FBG), lipid profiles including total cholesterol (TC), low density lipoprotein (LDL), high density lipoprotein (HDL) and triglycerides (TG) were all collected using standard protocols (33) and are described elsewhere.^9^

### Data analysis 

Statistical analyses were performed using Stata software version 14 (Stata Corp., College Station, TX). Linearized variance estimates were used to account for the complex survey design of NHANES. Four-year weights for the 2003 to 2006 survey periods were applied. Participant characteristics were presented as means with standard deviations (SD) (continuous variables) or as proportions (categorical variables). The total sample (n = 2143) was divided according to sex and stratified into RDW quartiles. Volume (how much SB and PA at different intensities) and pattern (sedentary and active bouts and breaks) were calculated and expressed using means and standard errors, and stratified according to RDW quartiles. Differences in characteristics across RDW quartiles were examined using ANOVA (continuous variables) and χ^2^ test (categorical variables). Statistically significant differences between groups were assumed based on non-overlapping confidence intervals (CI). Multivariable regression analyses were used to explore relationships between RDW and the accelerometer-derived variables. Significant covariates (*P* < 0.2) on univariate tests were included in multivariable analyses. The variance inflation factor was used to examine for collinearity between variables. R^2^ was calculated to determine the variability explained by the model. Statistical significance was set at α = 0.05.

## Results

### Basic characteristics of the study participants

Of the 20 470 participants, 4591 participants were identified within the selected age range and, of those, 2448 were excluded, due to missing RDW, PA or SB data. A total of 2143 children and adolescents aged from 12 to 20 years, mean age 15.3 (SD 2.2) were included. Demographic, anthropometric, clinical, laboratory, dietary, and PA intensities for boys and girls are presented according to RDW-Q status in Table 1. There was a significant difference across RDW quartiles for BMI, weight, WC, and mean SBP for girls. In addition, non-Hispanic/Black ethnicity and lower PIR were associated with higher RDW in girls (*P* < 0.001, and *P* = 0.011, respectively). Among boys, younger age, lower educational level, being non-Hispanic/Black and lower PIR were significantly associated with the highest RDW-Q compared to the lowest (*P* < 0.05). CRP was significantly associated with elevated RDW (*P* = 0.041) in boys but not in girls. Total caloric consumption and proportion of caloric intake by food type (i.e. carbohydrate, protein, and fat) were not significantly associated with RDW in both sexes, with the exception of the proportion of carbohydrates in boys (*P* < 0.047).

### Physical activity

Participants wore the accelerometer for an average of 5 days. Mean wear time was 14.8 (SD 2.3) and 14.5 (SD 2.1) hours per day for boys and girls, respectively. There were significant differences in SB, MPA, and VPA and in both average length of sedentary and active bouts between boys and girls (*P* < 0.001, for all), but not in LPA and sedentary breaks.

**Figure 1:**
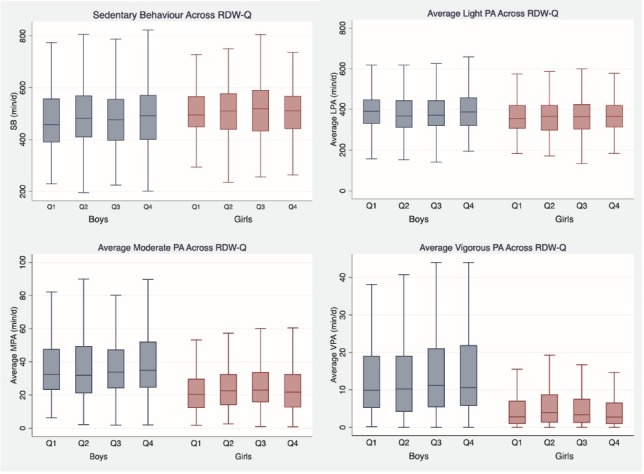
Sedentary behavior and physical activity across RDW-Q in boys and girls.
SB: sedentary behaviour; LPA: light physical activity; MPA: moderate physical activity; VPA: vigorous physical activity, RDW-Q: red blood cell distribution width quartile.

Volume of sedentary time and all PA intensities across RDW-Q in both boys and girls are presented in Figure 1. Boys in the highest RDW-Q spent an average of 498 minutes in SB, 39 minutes in LPA, 38 minutes in MPA, and 14 minutes in VPA compared to 487, 38, 37, and 13 minutes for boys in the lowest RDW-Q. Girls in the highest RDW-Q spent an average of 522 minutes in SB, 36 minutes in LPA, 24 minutes in MPA and 5 minutes in VPA compared to 516, 36, 22 and 5 minutes, for those in the lowest RDW-Q (Table 1). Although both boys and girls in the highest RDW-Q spent more sedentary time than those in lowest RDW-Q, the difference was not significant. Among girls, average MPA was significantly greater in the highest RDW-Q compared to the lowest, but lower than the second and third RDW-Q (*P* = 0.041). The associations between average sedentary bout length and number of sedentary breaks with RDW for both sexes were not significant.

### Regression analysis

Results of the multivariable regression analysis are presented in Table 2. In the fully adjusted model for boys, sedentary time (min/d) was significantly and positively associated with RDW levels (β = 0.116, *P* = 0.004, R^2^= 0.20). Time spent in MPA was significantly inversely associated with RDW (β = -0.082, *P* = 0.048, R^2^ = 0.19).The relationships between RDW, SB and PA among girls were not significant in any of the models. Furthermore, relationships with average sedentary bout length and number of sedentary breaks were not significant (data not shown).

## Discussion

The results of this study are consistent with previous studies with adults that reported associations between RDW, sedentary time and moderate PA.^9,18,25^ Though the underlying mechanism of the association between RDW and CVD remains unclear, there is strong evidence of a positive association between RDW and CVD and all-cause of CVD mortality in adults.^19,25,26^ In addition, previous studies with adults demonstrated that moderate-to-vigorous PA was independently associated with reduced odds of elevated RDW.^9,25^

Various theories exist about the mechanisms involved in the relationships between SB and PA and RDW. One of the mechanisms is via the influences of inflammation on anisocytosis.^25^ Prolonged SB increases inflammatory cytokines while PA has an anti-inflammatory effect. Inflammatory conditions target endothelium, and promote vascular dysfunction with subsequent atherosclerosis.^27^ It is unclear if RDW is an independent CVD risk factor or a marker of an underlying inflammatory state.^8^ However, in large studies, the association between higher RDW levels and CVD remained significant after adjusting for a number of confounding variables.^5,6^

For boys, the SB and MPA multivariable analysis explained nearly 20% and 19% of the variance of RDW, respectively. Although the magnitude of these estimated effects was smaller when compared to adults, it is important to consider the potential clinical significance of these findings. The stronger association in adults may be due to the accumulation of the effects of SB and PA over time. It is possible that increasing sedentary time may augment risks associated with elevated RDW, whereas increasing MPA may have a beneficial effect. From a public health perspective, this finding is important, as reducing sedentary time may be a particularly important message for boys. Further longitudinal and interventional research to examine the effects of changing SB and PA on CVD risk factors, including RDW, in children and adolescents is required.

Sedentary behavior has recently gained much interest as a risk factor for CVD morbidity and mortality.^11^ Developing findings suggest that increased SB is associated with CVD risk factors such as obesity, metabolic syndrome (MetS), and changes in lipoprotein lipase activity, independent of PA.^28^ Former studies in children and adolescents demonstrated associations between prolonged SB with MetS and cardiovascular health.^28^ Conversely, another study conducted on young children using NHANES database showed that diet and PA (using questionnaires) were not significantly associated with CVD risk factors or indicators of body fat.^29^ There is accumulating evidence that higher PA is strongly associated with a lower risk of CVD in adults and this relationship holds in young individuals.^30^ Increased PA in childhood can reduce CVD risks and may protect against atherosclerosis, if PA levels are maintained in young and middle adulthood.^30^

Increased SB and decreased PA were associated with higher RDW in boys and not girls. Sex differences in CVD disease risk have been demonstrated in children in other studies. For example, Füssenich et al,^31^ reported higher PA levels in boys, and also found significant differences in composite CVD risk scores between active and inactive boys but not in girls. In addition, previous research using NHANES data demonstrated that prevalence of CVD risk factors and MetS was higher in boys compared to girls.^32^ Increased prevalence of risk factors for boys appears to exist despite the fact that boys were more physically active and less sedentary.^21^

The difference between boys and girls is consistent with our knowledge regarding the later onset of CVD in women.^33^ Despite the fact that higher weight, BMI, WC, and SBP were associated with elevated RDW levels in girls but not in boys, relationships between the SB and PA variables and RDW were not significant. These results are consistent with previous work by Botton et al,^34^ who also found same similar sex-related associations between BMI, and WC with CVD risk factors. It is possible that CVD protective factors may attenuate the influence of PA levels on CVD risk factors, including RDW, in girls.^33^ Although CVD is a major cause of death in women, mean age of onset is 7-10 years later than men.^33^ Sex hormones may play a role attenuating the risk of CVD which could account for the sex-related differences in the relationships between SB, PA and RDW in this study.^35^ Other sex specific differences including different influences of CVD risk factors have been noted in the literature such as smoking, physical inactivity, dietary habits, MetS and emotional stress.^34^ These results further support sex specific analyses of risk factors related to CVD.

The cross-sectional design is a limitation of this study, since causality cannot be established. In addition, although the accelerometer is the most widely accepted way for assessing PA, accelerometry is limited by the inability to differentiate between different sedentary behaviour patterns which may not have the same association with health outcomes. Although we adjusted all models for potential confounders, the influence of latent variables especially sex-specific factors cannot be discounted.

## Conclusion

This study demonstrated an association between RDW, SB and MPA in boys, but not in girls. As the magnitude of differences were small, additional research may be needed to confirm these findings and their clinical significance, to clarify underlying mechanisms and to explore causal relationships. Prospective studies are also needed to evaluate the effects of activity modification on RDW levels and the potential influence on CVD risk. Future work in this area should consider sex-specific differences in children and adolescents.

## Ethical approval

No ethical approval was required for this study. NHANES data collection was approved by the ethics committee at the National Center for Health Statistics. Consent was obtained from all participants prior to data collection.

## Competing interests

The authors declare no potential conflicts of interest with respect to the research, authorship, and/or publication of this article.

## Authors’ contributions

All authors were involved in the conceptualization of the study. NH and VEE performed the data analysis and interpretation. NH drafted the manuscript and acted as corresponding author. TM and LPW supervised the development of work, assisted with data interpretation and manuscript revision.

## Acknowledgments

No funding was used to prepare this manuscript. Dr L.P.-W is supported by the Canadian Child Health Clinician Scientist Training Program (CCHCSP), the Women and Children’s Health Research Institute (WCHRI) through the generous support of the Stollery Children’s Hospital Foundation, and Alberta Policy Wise for Children and Families. VE is supported by Alberta Innovates Clinician Fellowship Award (#201600292).

**Table 1:** Basic characteristics of the study participants, by sex (n = 2143)

**Variables**	**Boys (n=1080)**					**Girls (n = 1063)**				
	**1st RDWQ (n= 261)**	**2nd RDWQ (n= 233)**	**3rd RDWQ (n = 303)**	**4th RDWQ (n = 283)**	***P *** **value**	**1st RDWQ (n = 264)**	**2nd RDWQ (n = 253)**	**3rd RDWQ (n = 259)**	**4th RDWQ (n = 287)**	*** P *** **value**
RDW%	11.7 ± 0.23	12.2 ± 0.07	12.5 ± 0.11	13.5 ±1.02	**0.000**	11.6 ± 0.21	12.1 ± 0.11	12.5 ± 0.13	13.8 ±1.09	**0.000**
Age (y)	15.5 ± 2.3	15.5 ± 2.2	15.2 ± 2.2	14.9 ± 2.2	**0.007**	15.1 ± 2.1	15.5 ± 2.2	15.2 ± 2.3	15.4 ± 2.3	0.119
**Education**										
<High school education	209 (81.3%)	186 (81.9%)	258 (87.8%)	254 (91.4%)	**0.011**	228 (86.3%)	213 (84.5%)	221 (86.7%)	236 (84.9%)	0.641
High school graduate	25 (9.7%)	25 (11%)	21 (7.1%)	11 (3.9%)		20 (7.6%)	18 (7.1%)	16 (6.3%)	27 (9.97%)
>High school education	23 (9 %)	16 (7.0%)	15 (5.1%)	13 (4.7%)		16 (6.1%)	21 (8.3%)	18 (7.1%)	15 (5.4%)
**Race/Ethnicity**										
Mexican American	91 (35%)	84 (36%)	114 (38%)	71 (25%)	**0.000**	85 (32%)	93 (37%)	101 (39%)	93 (32%)	**0.000**
Other Hispanic	6 (2.3%)	8 (3.4%)	10 (3.3%)	10 (3.5%)		6 (2.2%)	12 (4.7%)	4 (1.54%)	5 (1.7%)	
Non-Hispanic white	90 (34%)	77 (33%)	60 (20%)	25 (9%)		90 (34%)	74 (29%)	55 (21%)	36 (12%)	
Non-Hispanic Black	71 (27%)	50 (21%)	114 (38%)	165 (58%)		65 (25%)	66 (26%)	87 (34%)	144 (50%)	
Other, multi-racial	3 (1.1%)	14 (6%)	5 (1.6%)	12 (4.2%)		18 (6.8%)	8 (3.1%)	12 (4.6%)	9 (3.1%)	
**Family Poverty Income Ratio (PIR)** ^§^	2.2 ± 0.1	2.3 ± 0.1	2.1 ± 0.1	1.7 ± 0.1	**0.000**	2.3 ± 0.1	2.1 ± 0.1	1.9 ± 0.1	1.9 ± 0.1	**0.011**
Weight (kg)	66.8 ± 19	67.3 ± 17	67.9 ± 19	67.6 ± 21	0.937	57.9 ± 14	61.3 ± 16	61.2 ± 16	66.5 ± 20	**0.000**
Height (cm)	169.8 ± 11	170.4 ± 10	169.6 ± 10	167.9 ± 10	**0.045**	159.4 ± 7	159.7 ± 7	160.1 ± 6	160.6 ± 7	0.203
BMI (kg/m^2^)	22.8 ± 5	22.9 ± 4	23.3 ± 5	23.7 ± 6	0.217	22.6 ± 5	23.9 ± 6	23.8 ± 6	25.7 ± 7	**0.000**
Waist (cm)	80.2 ± 14	80.9 ± 12	80.9 ± 15	80.5 ± 16	0.936	78 ± 11	80 ± 14	81 ± 14	84.3 ± 16	**0.000**
SBP (mmHg)	112 ± 10	112 ± 10	112.3 ± 10	113 ± 10	0.638	106.3 ± 9	106 ± 9	106 ± 10	108 ± 9	**0.027**
DBP (mmHg)	78 ± 19	78 ± 16	79 ± 18	79 ± 19	0.886	78.8 ± 14	78.2± 14	77.8 ± 14	78 ± 15	0.859
**Laboratory Markers**										
Cotinine (ng/mL) ^§^	16.4 ± 4	19.8 ± 5	10.3 ± 2	15.4 ± 4	0.307	7 ± 2.3	8.3 ± 2.3	11.8 ± 3	7.4 ± 2	0.478
Hemoglobin (g/dL)	15.2 ± 1.2	15.1 ± 1.0	14.9 ± 1.0	13.3 ± 1.2	**0.000**	13.7 ± 0.8	13.6 ± 0.8	13.4 ± 0.9	12.6 ± 1.1	**0.000**
Serum iron (µg/dL)	102 ± 38.2	98 ± 36.8	91 ± 37.1	81 ± 34.6	**0.000**	88 ± 34	83 ± 36	77 ± 35	63 ± 32	**0.000**
CRP (mg/dL) ^§^	0.12 ± 0.01	0.11 ± 0.01	0.15 ± 0.02	0.19 ± 0.02	**0.041**	0.15 ± 0.02	0.2 ± 0.04	0.25 ± 0.06	0.28 ± 0.03	0.141
Uric acid (mg/dL)	5.4 ± 1.1	5.5 ± 1.0	5.6 ± 1.1	4.4 ± 1.1	0.084	4.4 ± 0.8	4.3 ± 0.8	4.4 ± 1	4.3 ± 1	0.681
LDL-C (mmol/L)	2.3 ± 0.67	2.3 ± 0.68	2.3 ± 0.68	2.2 ± 0.70	0.602	2.3 ± 0.64	2.3 ± 0.57	2.3 ± 0.75	2.3 ± 0.65	0.821
HDL-C (mmol/L)	1.3 ± 0.30	1.3 ± 0.29	1.3 ± 0.32	1.4 ± 0.33	0.096	1.4 ± 0.32	1.4 ± 0.33	1.4 ± .35	1.4 ± 0.34	0.966
Total-C (mmol/L)	4.1 ± 0.74	4.1 ± 0.77	4.1 ± 0.73	4.1 ± 0.79	0.742	4.2 ± 0.80	4.2 ± .63	4.2 ± 0.78	4.2 ± 0.76	0.890
TG (mmol/L)^§^	1.0 ± 0.04	1.0 ± 0.05	0.95 ± 0.04	0.92 ± 0.03	**0.010**	0.9 ± 0.04	0.9 ± .04	0.8 ± 0.03	0.9 ± 0.03	0.997
FBS (mg/dL)	94.7 ± 18	93.2 ± 7.6	93.3 ± 8.3	92.8 ± 7.5	0.547	91.1 ± 10	98.3 ± 7.6	90.5 ± 7.7	89.3 ± 9.7	0.315
**Diet Components**										
Energy (kcal)	5043 ± 1788	5092 ± 1823	4854 ± 1806	4823 ± 1803	0.541	3920 ± 1295	3892 ± 1425	3733 ± 1328	4007 ± 1452	0.140
% Energy from carb.	0.52 ± 0.08	0.54 ± 0.07	0.52 ± 0.08	0.53 ± 0.07	**0.047**	0.53 ± 0.08	0.54 ± 0.07	0.52 ± 0.08	0.53 ± 0.07	0.210
% Energy from protein	0.26 ± 0.06	0.25 ± 0.05	0.25 ± 0.06	0.25 ±0.06	0.507	0.26 ± 0.06	0.25 ± 0.05	0.25 ± 0.06	0.25 ± 0.06	0.225
% Energy from fat	0.33 ± 0.06	0.32 ± 0.06	0.33 ± 0.06	0.33 ± 0.05	0.079	0.33 ± 0.06	0.32 ± 0.06	0.33 ± 0.06	0.33 ± 0.05	0.390
**Physical Activity Variables**										
Wear time (min/d)	881 ± 135	883 ± 138	886 ± 129	904 ± 144	0.189	866 ± 118	872 ± 132	878 ± 128	878 ± 133	0.693
Valid days (N)	5.5 ± 1	5.7 ± 1	5.6 ± 1	5.7 ± 1	0.281	5.6 ± 1	5.6 ± 1	5.6 ± 1	5.6 ± 1	0.924
SB (min/d) ^§^	487 ± 8.2	500 ± 8.8	492 ± 7.6	498 ± 8.8	0.684	516 ± 7.1	517 ± 7.6	521 ± 8.3	522 ± 7.4	0.925
Breaks in ST (total number)^§^	538 ± 9	545 ± 9	530 ± 8	555 ± 9	0.227	537 ± 8	546 ± 10	541 ± 10	542 ± 9	0.932
Average sedentary bout length (min/d)^§^	5.1 ± 0.09	5.3 ± 0.1	5.3 ± 0.08	5.2 ± 0.09	0.564	5.4 ± 0.07	5.5 ± 0.1	5.5 ± 0.09	5.5 ± .08	0.955
Average active bout length (min/d)^§^	4.3 ± 0.11	4.1 ± 0.06	4.2 ± 0.06	4.3 ± 0.9	0.238	3.7 ± 0.08	4 ± 0.16	3.8 ± 0.07	3.7 ± 0.9	0.559
Average LPA (min/d)^§^	38.7 ± 5.6	37.4 ± 6.1	38.5 ± 5.6	39.1 ± 5.7	0.218	36.5 ± 5.2	36.9 ± 5.8	36.8 ± 5.3	36.7 ± 5	0.957
Average MPA (min/d)^§^	36.9 ± 1.1	35.7 ± 1.3	37 ± 1	38.4 ± 1.2	0.435	22.4 ± 0.8	25.1 ± 1	26 ± 0.9	24.2 ± 0.9	**0.041**
Average VPA, (min/d)^§^	13.7 ± 0.7	14.1 ± 0.8	14.8 ± 0.7	14.5 ± 0.7	0.720	5.3 ± 0.4	6.4 ± 0.5	6 ± 0.5	5.6 ± 0.5	0.337

RDW: red blood cell distribution width; BMI: body mass index; SBP: systolic blood pressure; DBP: diastolic blood pressure; CRP: C-reactive protein; LDL-C: low density lipoprotein-cholesterol; HDL-C: high density lipoprotein-cholesterol; Total-C: total cholesterol; TG: triglyceride; FBS: fasting blood glucose; carb.: carbohydrate; SB: sedentary behaviour; ST: sedentary time, LPA: light physical activity; MPA: moderate physical activity; VPA: vigorous physical activity; means ± SD for continuous variables or N. (%) for categorical variables, ST= sedentary time, § (mean ±SE)

**Table 2:** Linear regression analysis for RDW, sedentary behaviour and physical activities

**Variables**	**Boys**				**Girls**			
	**1st Model**	**2nd Model**	**3rd Model**	**4th Model**	**1st Model**	**2nd Model**	**3rd Model**	**4th Model**
SB								
β	0.050	0.019	0.026	0.116	-0.004	-0.026	-0.023	-0.017
*P* value	0.100	0.512	0.345	**0.004**	0.889	0.382	0.378	0.507
R^2^	0.001	0.096	0.189	0.200	-0.000	0.055	0.254	0.263
LPA								
β	0.048	0.030	0.005	0.025	0.034	0.023	0.026	0.017
*P* value	0.111	0.302	0.847	0.525	0.257	0.444	0.339	0.527
R^2^	0.001	0.096	0.188	0.187	0.000	0.055	0.254	0.263
MPA								
β	0.031	-0.026	-0.051	**-**0.082	0.012	0.014	0.012	0.016
*P* value	0.297	0.381	0.071	**0.048**	0.689	0.645	0.646	0.542
R^2^	0.000	0.096	0.191	0.193	0.000	0.055	0.253	0.263
VPA								
β	0.025	-0.016	-0.022	-0.060	-0.041	-0.019	-0.008	0.002
*P* value	0.417	0.579	0.434	0.133	0.181	0.515	0.751	0.952
R^2^	0.000	0.096	0.189	0.190	0.000	0.055	0.253	0.263

SB: sedentary behaviour; LPA: light physical activity; MPA: moderate physical activity; VPA: vigorous physical activity; β: standardized bata; R2: adjusted R square. The 1st model: unadjusted model; the 2nd model was adjusted for demographics: age, gender and ethnicity, the 3rd model was adjusted for the CRP and HGB in addition to the covariates described in model 2 and the 4th model (full adjusted model) was adjusted for BMI and HDL-C in addition to the co-variates in model 3.
